# Prophylactic mesh augmentation after laparotomy for elective and emergency surgery: meta-analysis

**DOI:** 10.1093/bjsopen/zrad060

**Published:** 2023-07-28

**Authors:** Simone Frassini, Francesca Calabretto, Stefano Granieri, Paola Fugazzola, Matteo Massaro, Benedetta Sargenti, Luca Schiavone, Simone Zanghì, Francesca Dal Mas, Luca Ansaloni, Lorenzo Cobianchi

**Affiliations:** General Surgery Residency, University of Pavia, Pavia, Italy; Unit of General Surgery I, Fondazione I.R.C.C.S. Policlinico San Matteo, Pavia, Italy; General Surgery Residency, University of Pavia, Pavia, Italy; Unit of General Surgery I, Fondazione I.R.C.C.S. Policlinico San Matteo, Pavia, Italy; General Surgery Unit, ASST-Brianza, Vimercate Hospital, Vimercate, Italy; General Surgery Residency, University of Pavia, Pavia, Italy; Unit of General Surgery I, Fondazione I.R.C.C.S. Policlinico San Matteo, Pavia, Italy; General Surgery Residency, University of Pavia, Pavia, Italy; Unit of General Surgery I, Fondazione I.R.C.C.S. Policlinico San Matteo, Pavia, Italy; General Surgery Residency, University of Pavia, Pavia, Italy; Unit of General Surgery I, Fondazione I.R.C.C.S. Policlinico San Matteo, Pavia, Italy; General Surgery Residency, University of Pavia, Pavia, Italy; Unit of General Surgery I, Fondazione I.R.C.C.S. Policlinico San Matteo, Pavia, Italy; General Surgery Residency, University of Pavia, Pavia, Italy; Unit of General Surgery I, Fondazione I.R.C.C.S. Policlinico San Matteo, Pavia, Italy; Department of Management, Università Ca’ Foscari, Venezia, Italy; General Surgery Residency, University of Pavia, Pavia, Italy; Unit of General Surgery I, Fondazione I.R.C.C.S. Policlinico San Matteo, Pavia, Italy; General Surgery Residency, University of Pavia, Pavia, Italy; Unit of General Surgery I, Fondazione I.R.C.C.S. Policlinico San Matteo, Pavia, Italy

## Abstract

**Background:**

Incisional hernia is a common short- and long-term complication of laparotomy and can lead to significant morbidity. The aim of this systematic review and meta-analysis is to provide an up-to-date overview of the laparotomy closure method in elective and emergency settings with the prophylactic mesh augmentation technique.

**Methods:**

The Scopus, PubMed, and Web of Science databases were screened without time restrictions up to 21 June 2022 using the keywords ‘laparotomy closure’, ‘mesh’, ‘mesh positioning’, and ‘prophylactic mesh’, and including medical subject headings terms. Only RCTs reporting the incidence of incisional hernia and other wound complications after elective or emergency midline laparotomy, where patients were treated with prophylactic mesh augmentation or without mesh positioning, were included. The primary endpoint was to explore the risk of incisional hernia at different follow-up time points. The secondary endpoint was the risk of wound complications. The risk of bias for individual studies was assessed according to the Revised Cochrane risk-of-bias tools for randomized trials.

**Results:**

Eighteen RCTs, including 2659 patients, were retrieved. A reduction in the risk of incisional hernia at every time point was highlighted in the prophylactic mesh augmentation group (1 year, risk ratio 0.31, *P* = 0.0011; 2 years, risk ratio 0.44, *P* < 0.0001; 3 years, risk ratio 0.38, *P* = 0.0026; 4 years, risk ratio 0.38, *P* = 0.0257). An increased risk of wound complications was highlighted for patients undergoing mesh augmentation, although this was not significant.

**Conclusions:**

Midline laparotomy closure with prophylactic mesh augmentation can be considered safe and effective in reducing the incidence of incisional hernia. Further trials are needed to identify the ideal type of mesh and technique for mesh positioning, but surgeons should consider prophylactic mesh augmentation to decrease incisional hernia rate, especially in high-risk patients for fascial dehiscence and even in emergency settings.

**PROSPERO registration ID:**

CRD42022336242 (https://www.crd.york.ac.uk/prospero/record_email.php).

## Teaser

Incisional hernia is a common brief and long-term complication of laparotomy and can lead to significant morbidity. Our systematic review and meta-analysis aimed to provide an up-to-date overview of the laparotomy closure method in elective and emergency settings with the prophylactic mesh augmentation technique. Midline laparotomy closure with prophylactic mesh augmentation can be considered safe and effective in reducing the incidence of incisional hernia.

## Introduction

The optimal method for the closure of the abdominal wall after a midline laparotomy is still unclear and debated, despite recent guideline statements^1,2^. Acute wound dehiscence has a reported incidence ranging from 2 to 5.5 per cent across studies—typically occurring between postoperative day 6 and 12. The mortality in these cases may reach 20.9 per cent^3^.

Incisional hernia (IH) is a common short- and long-term complication of laparotomy, and can lead to significant morbidity, including pain, deformity, hospital re-admission, and re-operation^4–6^. The increased incidence of IH depends on patients’ risk factors, like advanced age, smoking, diabetes, high BMI, malnutrition, and pharmacologic use of corticosteroids and immunosuppressants. Surgery-related risk factors for IH include wound contamination, type of incision, type of surgery, and suture technique^7,8^.

The management of IH leads to both direct and indirect costs due to the nurse care and support services requirement, prolonged hospitalization, the use of wound dressings, and social support requirements. The combination of these factors has a burdensome impact on the healthcare system^9,10^. Moreover, the repair of fascial defects can be a complex surgical challenge, mostly in frail patients, characterized by a high rate of failure and common recurrence^11^.

The use of prophylactic mesh placement after midline laparotomy is a technique of great interest, with promising results in several RCTs. Despite the results of some of these studies, many surgeons remain concerned about mesh use due to the risk of infection, complications, increased surgical time, and cost of materials^12^. In addition, recently published guidelines by the European Hernia Society state that the fascia should be closed directly using a continuous suturing technique, based on outcomes after elective laparotomy, and a prophylactic mesh augmentation can be suggested with a weak recommendation only in high-risk patients^1,2^. The ‘HERNIA PROJECT’ states that high-risk conditions for IH development are diabetes, chronic pulmonary disease, smoking, obesity, immunosuppression, surgical site infections, and previous abdominal surgery^13^. On the other hand, none of the previously published studies had examined which kind of mesh positioning could be the ideal one after midline laparotomy, and data are lacking about this technical detail, especially in emergency settings^14,15^.

This systematic review and meta-analysis aims to provide an up-to-date overview of laparotomy closure method in elective and emergency settings with the prophylactic mesh augmentation technique.

## Methods

### Search strategy

A systematic review of literature in the English language was performed according to the Preferred Reporting Items for Systematic Reviews and Meta-Analyses (PRISMA) and AMSTAR (Assessing the methodological quality of systematic reviews) guidelines^16–18^, and the Meta-Analyses and Systematic Reviews of Observational Studies (MOOSE) recommendations^18^. The systematic review protocol was registered on the International Prospective Register of Systematic Reviews—PROSPERO (registration ID: CRD42022336242).

The Scopus, PubMed, and Web of Science databases were screened without time restrictions up to 21 June, 2022 using the keywords ‘laparotomy closure’, ‘mesh’, ‘mesh positioning’, and ‘prophylactic mesh’, and including medical subject headings (MeSH) terms. Articles without available free full text were searched through the University of Pavia and University of Milan digital library, and direct contact with authors. Additional relevant studies were hand-searched throughout the reference lists of all included studies and previous reviews on the topic. Two investigators (S.F. and F.C.) carried out the literature search independently. After the exclusion of duplicates, two independent reviewers (S.F. and F.C.) screened titles and abstracts. Investigators were blinded to each other’s decisions. Any disagreement was solved by the senior authors (L.A. and L.C.).

### Inclusion criteria

Only RCTs reporting the incidence of IH and other wound complications after elective or emergency midline laparotomy, where patients were treated with prophylactic mesh augmentation or without mesh positioning, were included.

A specific population (P), intervention (I), comparator (C), outcome (O), and study design (S) (PICOS) framework was specified to define study eligibility, as recommended. In particular:

- Population (P): adult patients undergoing elective or emergency laparotomy;
- Intervention (I): laparotomy closure with prophylactic mesh augmentation;
- Comparison (C): laparotomy closure without prophylactic mesh augmentation;
- Outcomes (O): incidence of IH and other wound complications;
- Study design (S): RCTs only.

Studies with insufficient reporting of the PICOS criteria were excluded.

### Exclusion criteria

All non-randomized studies were excluded from the present review. Case reports, preclinical and non-human studies, meta-analyses, editorials, previous reviews, book chapters, and commentaries were considered not eligible for analysis. Studies written in languages other than English were also excluded. Among trials from the same institution reporting results from potentially overlapping series, only the most recent or the one with the most accurate and complete data reporting was selected.

PRISMA, AMSTAR-2, and MOOSE checklists are reported in the *Supplementary material*.

### Assessment of risk of bias

The risk of bias for individual studies was assessed according to the Revised Cochrane risk-of-bias tools for randomized trials (RoB 2)^19^ independently by two investigators (S.F. and M.M.).

Data were collected according to the methodology proposed by Higgins^20^ in a computerized spreadsheet. The results of the risk-of-bias assessment were graphically reported as bar and traffic light plots.

### Data extraction and assessment of included studies

Data were extracted independently by four authors (B.S., M.M., L.S., and S.Z.). Information about study design and methodology, participant demographic characteristics, laparotomy indication and surgical technique, type of intervention (elective or emergency), prophylactic use of mesh or traditional closure technique, IH rate, and wound failure rate were gathered in a computerized spreadsheet (Microsoft Excel 2016; Microsoft Corporation, Redmond, WA, USA). In case of disagreement, two further investigators (S.F. and F.C.) helped resolve it through discussion.

### Primary and secondary endpoints

The primary endpoint was represented by the incidence of IH after midline laparotomy at 1-, 2-, 3- and 4-year follow-up. The secondary endpoint was to investigate the incidence of other wound complications, including wound dehiscence, wound infections, and mortality.

### Statistical analysis

The risk ratio (RR) and the relative 95 per cent confidence intervals (c.i.) were adopted as the primary and secondary outcome measures. Meta-analyses of binary outcomes were built. The results were reported as a whole and stratified by subgroups (elective/emergency surgery). Fixed- and random-effects models based on the Mantel–Haenszel method were built to assess the impact of heterogeneity on results. In the presence of low heterogeneity (<25 per cent), a fixed-effects model was chosen to compute the outcome. The presence of outliers was explored, and their effect sizes were excluded.

Heterogeneity between studies was quantified by the *I*^2^ statistic and Cochran’s Q test; cut-off values of 25 per cent, 50 per cent, and 75 per cent were considered as low, moderate, and high respectively^20^. Sensitivity analyses were conducted after inspecting patterns of effect sizes and heterogeneity of the included studies. To identify studies overly contributing to heterogeneity, Graphic Display of Heterogeneity (GOSH) plots were developed and sensitivity analysis was conducted, excluding studies predominantly responsible for heterogeneity.

Funnel plots were developed to explore publication bias, and Egger’s test of the intercept was used to quantify funnel plot asymmetry. Duval and Tweedie’s trim-and-fill method was adopted to estimate and adjust for the number and outcomes of missing studies each time Egger’s test demonstrated significant asymmetry.

Statistical analysis was conducted using R statistical software (The Comprehensive R Archive Network—CRAN, ver. 4.0.0 ×64)^21^, using ‘meta’, ‘metafor’, ‘robvis’, and ‘dmetar’ packages^21–24^.

## Results

### Descriptive non-comparative analysis of included studies and primary endpoint

**Fig. 1:**
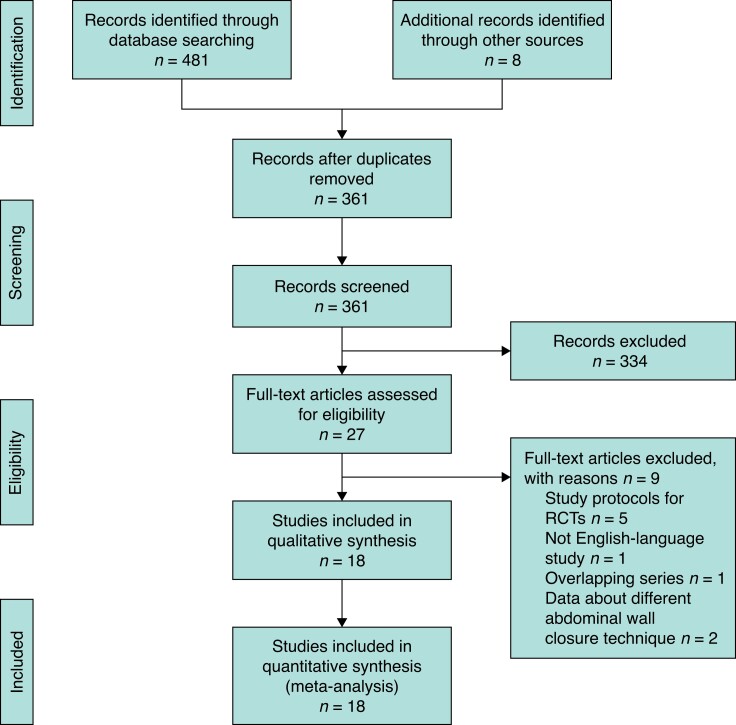
PRISMA flow diagram

Overall, 489 articles were preliminarily identified by the literature search. After the exclusion of duplicates, the titles and abstracts of 361 records were screened. Twenty-seven full-text articles were assessed for eligibility. After the literature selection, 18 RCTs^25–41^ were included in the qualitative and quantitative analysis (*Fig. 1*).

The studies by Brosi^38^ and Glauser Philippe^39^ refer to the same case series but reported different outcomes; the same applies to the studies published by Caro-Tarrago in 2014 and 2019^40,41^. Since the studies reported different outcome data, they were all included.

In total, 2659 patients were included in the meta-analysis. All studies came from Western countries. Some 1403 patients underwent abdominal wall closure with prophylactic mesh augmentation, whereas 1156 were treated without any mesh; in emergency settings, 185 were treated with mesh placement and 173 without mesh placement. *Table 1* summarizes patients’ characteristics.

**Table 1:** Detailed characteristics of included studies

Author	Years of enrollment	Patients enrolled (*n*)	Mesh group (*n*)	Control group (*n*)	Elective surgery		Emergency surgery		Follow-up time (years)
					Mesh	Control	Mesh	Control	
Pans *et al.*^26^	1990–1993	288	144	144	144	144	0	0	2
Gutiérrez de la Peña *et al.*^27^	1998–2001	100	50	50	50	50	0	0	3
Strzelczyk *et al.*^28^	2002–2005	74	36	38	36	38	0	0	2
El-Khadrawy *et al.*^29^	2000–2002	40	20	20	20	20	0	0	3
Bevis *et al.*^30^	2003–2007	80	37	43	37	43	0	0	3
Abo-Ryia *et al.*^31^	2004–2006	64	32	32	32	32	0	0	4
Bali *et al.*^32^	2007–2009	40	20	20	20	20	0	0	3
Sarr *et al.*^33^	2005–2012	380	139	141	139	141	0	0	2
Garcia-Urena *et al.*^34^	2009–2011	107	53	54	33	37	20	17	2
Muysoms *et al.*^35^	2009–2013	114	56	58	56	58	0	0	2
Jairam *et al.*^36^	2009–2012	480	373	107	373	107	0	0	2
Kohler *et al.*^6^	2011–2014	150	69	81	69	81	0	0	4
Lima *et al.*^37^	2015–2018	115	63	52	0	0	63	52	1
Brosi *et al.*^38^ and Glauser *et al.*^39^	2008–2013	267	131	136	129	132	2	4	5
Caro-Tarrago *et al.*^40,41^	2009–2012	160	80	80	80	80	0	0	5
Pizza *et al.*^42^	2015–2018	200	100	100	0	0	100	100	2

### Primary endpoint

A significant reduction in the risk of IH at every time point was highlighted in the prophylactic mesh augmentation group. Meta-analysis of binary outcomes pointed out at 1-, 2-, 3-, and 4-year follow-up an overall RR with random-effects model of 0.31 (95 per cent c.i. 0.16–0.63; *I*^2^ = 64.5 per cent; *P* = 0.0011), 0.44 (95 per cent c.i. 0.32 to 0.64; *I*^2^ = 52.7 per cent; *P* < 0.0001), 0.38 (95 per cent c.i. 0.21 to 0.72; *I*^2^ = 42.6 per cent; *P* = 0.0026), and 0.38 (95 per cent c.i. 0.17 to 0.89; *I*^2^ = 75.8 per cent; *P* = 0.0257) respectively.

Subgroup analysis at 1- and 2-year follow-up comparing elective and emergency surgery showed a greater risk reduction in the emergency surgery subgroup without reaching statistical significance in the random-effects model (RR 0.24, 95 per cent, c.i. 0.09 to 0.61, *P* = 0.4670 at 1 year; RR 0.34, 95 per cent, c.i. 0.18 to 0.64, *P* = 0.4007 at 2 years). Conversely, at 4-year follow-up, the elective surgery subgroup reported a greater risk reduction (RR 0.33, 95 per cent, c.i. 0.12 to 0.93, *P* = 0.4560).

**Fig. 2:**
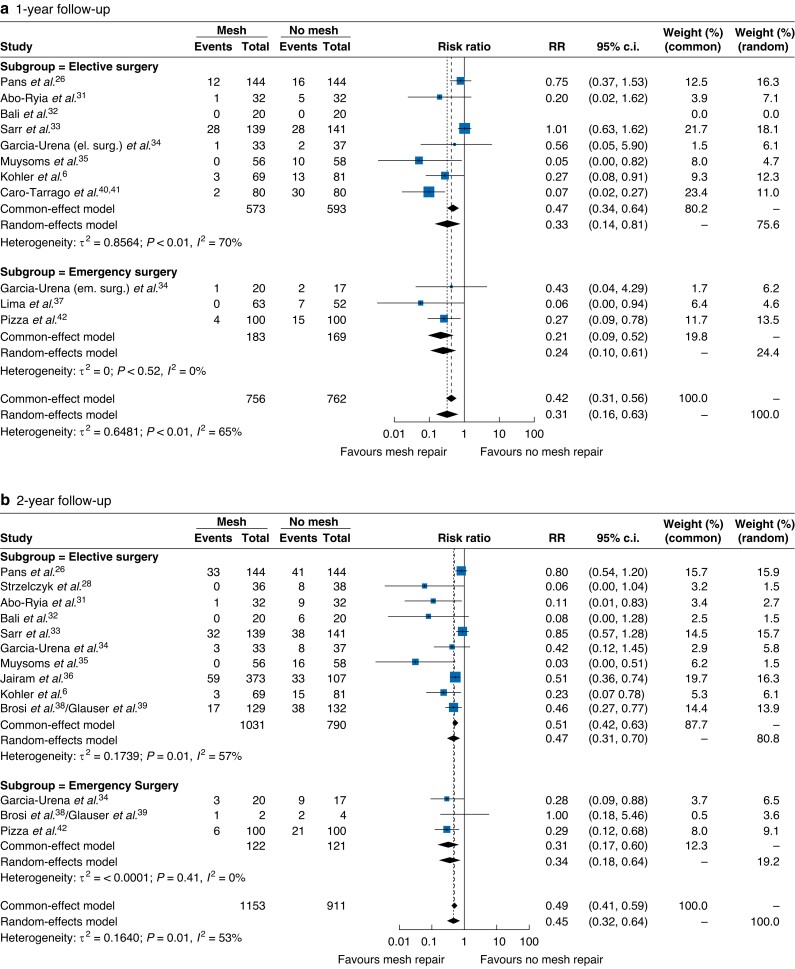

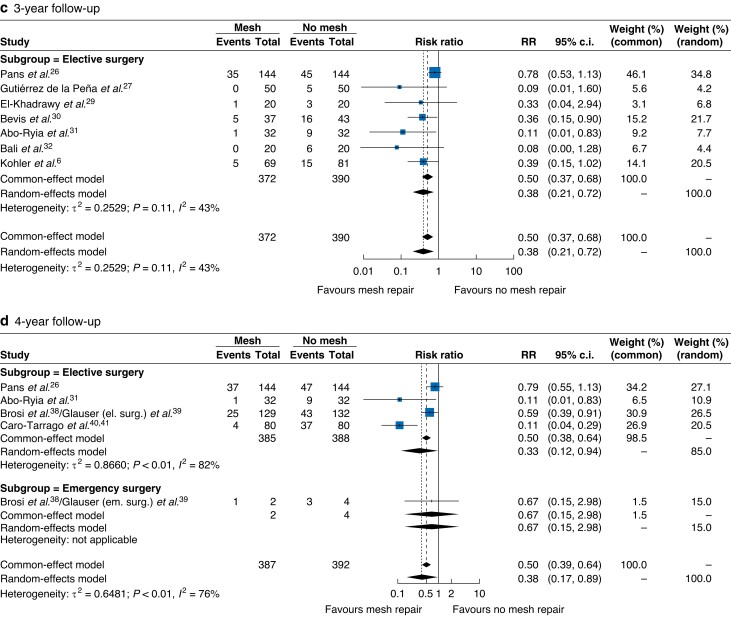
Forest plots of primary endpoint (incidence of incisional hernia) at a) 1-year, b) 2-year, c) 3-year, and d) 4-year follow-up

Forest plots of primary endpoints are shown in *Fig. 2*.

#### Sensitivity analysis

**Fig. 3:**
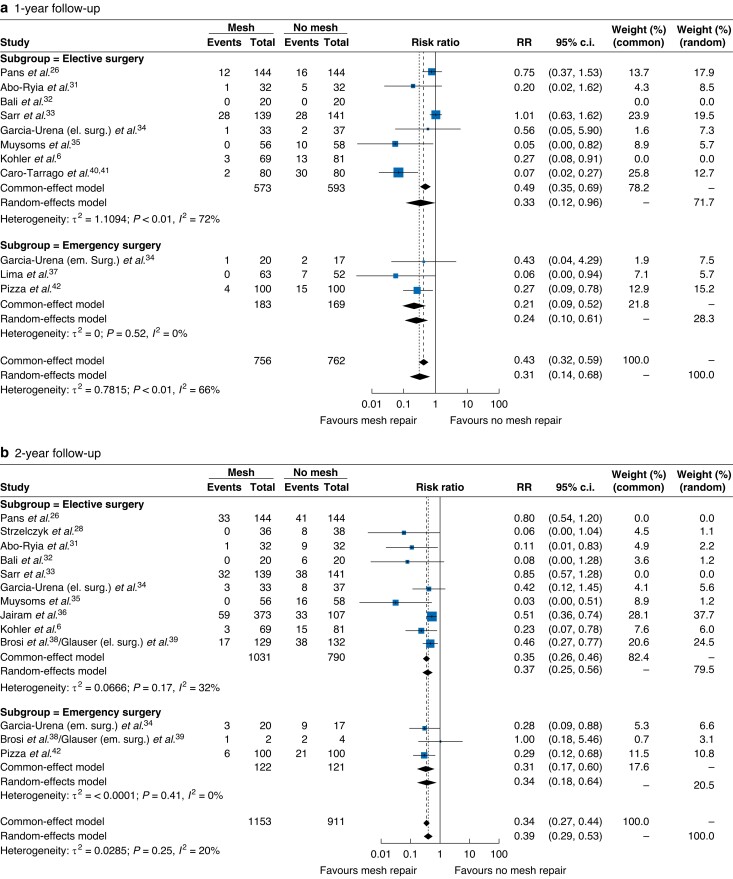

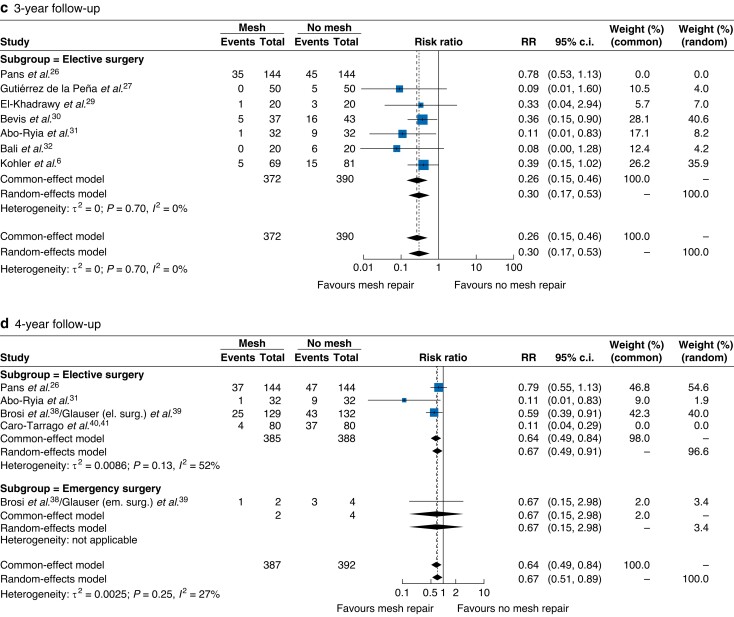
Forest plots for incisional hernia at a) 1-year, b) 2-year, c) 3-year, and d) 4-year follow-up after sensitivity analysis

After GOSH plot assessment and identification of outliers, sensitivity analysis was conducted. Ten studies^6,25,30–34,36,39,41^ reported data at 1-year follow-up. After excluding the studies by Bali and Kohler^6,32^, a significant IH risk reduction was confirmed for mesh patients (*Fig. 3a*, RR 0.31; 95 per cent c.i. 0.14 to 0.68; *P* < 0.001; *I*^2^ = 66 per cent). Subgroup analysis confirmed a reduced risk of IH for these patients in the emergency setting (RR 0.21; 95 per cent c.i. 0.09 to 0.52; *P* = 0.2421).

Eleven studies^25,27,30–37,41^ reported data at 2-year follow-up. After excluding the studies by Pans and Sarr^26,33^, a significant overall effect was confirmed for patients receiving mesh augmentation (*Fig. 3b*, RR 0.34, 95 per cent c.i. 0.27 to 0.44; *P* < 0.01; *I*^2^ = 20.3 per cent). This finding was also confirmed in subgroup analysis for patients operated on in an emergency setting (RR 0.31; 95 per cent c.i. 0.17 to 0.60; *P* = 0.7717).

At 3-year follow-up, seven studies reported data regarding IH^24,25,27–30,35^. Only the study by Pans^26^ was detected as overtly contributing to heterogeneity. After excluding it, an overall effect was confirmed for patients undergoing mesh augmentation (*Fig. 3c*, RR 0.26; 95 per cent c.i. 0.15 to 0.46; *P* < 0.0001; *I*^2^ = 0 per cent). No subgroup analysis was conducted due to the lack of emergency cases.

At 4-year follow-up, only four studies^25,30,38,40^ reported information regarding the primary outcome. After excluding the study by Caro-Tarrago^40^, an overall effect was confirmed for patients receiving mesh augmentation (*Fig. 3d*, RR 0.67; 95 per cent c.i. 0.51 to 0.89; *P* = 0.0013; *I*^2^ = 27.3 per cent). Only one study^38^ reported data regarding emergency surgery. The risk of IH was comparable in both elective and emergency patients.

### Secondary endpoint

#### Wound dehiscence

**Fig. 4:**
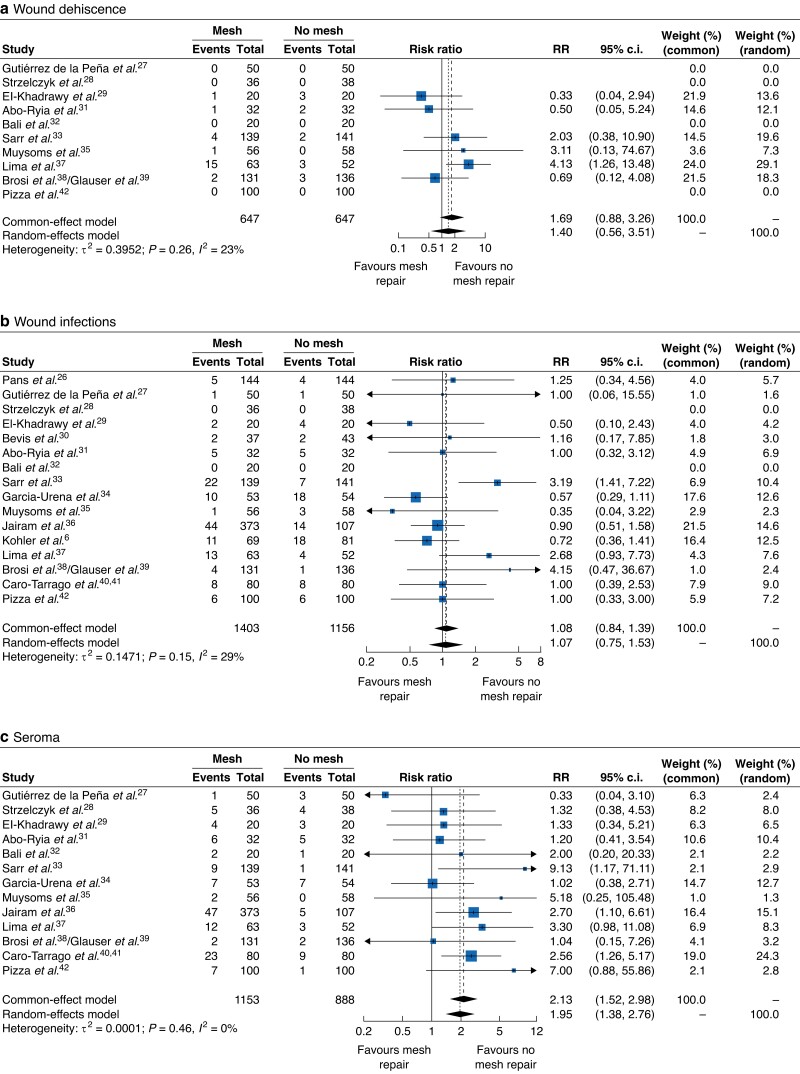
Forest plots of a) wound dehiscence, b) wound infection, c) seroma

Meta-analysis of binary outcomes of 10 studies^27–29,31–33,35,37,39,42^ showed no significant augmented risk of wound dehiscence for patients undergoing abdominal wall mesh repair (RR 1.69; 95 per cent c.i. 0.88 to 3.26; *P* = 0.1175; *I*^2^ = 23.1 per cent. *Fig. 4a*).

#### Wound infection

Sixteen studies^6,26–37,39,41,42^ reported data regarding wound infections. Patients undergoing mesh augmentation showed a slight, non-significant increase in the risk of surgical site infection (SSI) (RR 1.07; 95 per cent c.i. 0.75 to 1.53; *P* = 0.5321; *I*^2^ = 28.7 per cent; *Fig. 4b*).

#### Seroma

Meta-analysis of binary outcomes of 13 studies^27–37,39,41,42^ showed an increase in the risk of seroma development for patients treated with mesh augmentation, and statistical significance was highlighted (RR 2.13; 95 per cent c.i. 1.52 to 2.98; *P* < 0.0001; *I*^2^ = 0 per cent; *Fig. 4c*).

### Risk-of-bias assessment

**Fig. 5:**
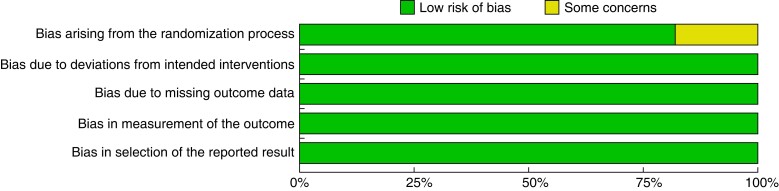
Risk of bias assessment through barplot

The risk-of-bias assessment showed a low risk of bias in all domains of all included studies except in ‘Bias arising from the randomization process’, with ‘some concerns’ in the study published by Jairam *et al.*^36^ (*Fig. 5*).

### Assessment of publication bias

Egger’s regression test of IH pointed out significant asymmetry at all time points (1 year *P* = 0.0005, 2 years *P* = 0.0004, 3 years *P* = 0.0022), except the 4-year follow-up (*P* = 0.183). Funnel plots of publication bias were developed for a graphical assessment of the publication bias.

## Discussion

Postoperative IH is a critical health issue and can result in high morbidity and even mortality when complications occur. In addition, patients with peritonitis undergoing emergency laparotomies have an increased risk of IH and wound dehiscence compared with patients treated in elective settings. Other patient risk factors for IH—according to the prospective study by Goodenough *et al*.—are diabetes, chronic pulmonary disease, smoking, obesity, and immunosuppression^13^.

A Belgian research group was the first in 1995 to publish results about prophylactic mesh augmentation in the closure of abdominal wall incisions to reduce the incidence of IH^43^. Since then, multiple RCTs have been published confirming the efficacy of the prophylactic mesh augmentation technique: data highlights a significant reduction of IH in elective surgery but even a significantly lower rate of IH when emergency laparotomy is performed^42^. On the other hand, many surgeons are still concerned about mesh reinforcement in the case of contaminated surgical fields, specifically considering the risk of infection and other postoperative complications. Nevertheless, no increase in postoperative complications, except for a greater incidence of seroma, has been found when the prophylactic mesh augmentation technique is performed^12^.

The present study was designed to evaluate the efficacy of the prophylactic mesh positioning technique compared with traditional abdominal closure after midline laparotomy, in both elective and emergency/trauma surgery settings. Compared with other systematic reviews and meta-analyses already available^12,44–50^, this article encompasses all RCTs published to date involving the largest population available in the literature and also focusing on patients undergoing emergency laparotomies.

The results from this study highlighted the beneficial role of prophylactic mesh augmentation for the closure of abdominal wall incision; regarding the primary endpoint, a significant reduction in the risk of IH was evidenced at every time point of follow-up. Subgroup analysis showed a significant risk reduction at 1- and 2-year follow-up when prophylactic mesh augmentation was performed in the emergency setting. This trend was not confirmed at 4-year follow-up when a higher IH risk reduction was pointed out for patients undergoing elective surgery. On the other hand, patients without prophylactic mesh placement showed a lower incidence of wound dehiscence, wound infections, and seroma. Data regarding the secondary outcomes found that no additional complications were significantly related to prophylactic mesh positioning, except for seroma formation.

Nevertheless, some aspects of the results need to be examined in more detail. From 2013, multiple reviews and meta-analyses have examined this topic with substantial accordance about the beneficial role of mesh placement in preventing IH, but they were mainly focused on elective surgery. Tiemermans *et al*. and Bhangu *et al.* stated that the mesh augmentation technique decreases the IH rate compared with the standard technique in elective high-risk patients, but the authors identified poor assessment of other outcomes and some limits in applicability^5,44^. Some additional evidence, concordant with the beneficial role of mesh positioning, came from further studies in elective specialist surgery: Dasari *et al.* in 2016 analysed outcomes in bariatric surgery, Indrakusuma *et al.* in 2018 and Nicolajsen *et al.* in 2020 focused on laparotomy closure after open treatment of aortic aneurysm^5,7^. Conversely, a lack of evidence about outcomes in emergency settings emerged from the literature. For example, in 2016 Borab *et al*. published a review regarding laparotomy closure without data about emergency laparotomies, which were considered as an exclusion criterion in the selection process^46^. Payne *et al.* in 2017 confirmed that the use of prophylactic mesh significantly reduces the occurrence of IH after laparotomy both in elective and emergency surgery without subgroup analysis, meanwhile, a publication by Burns *et al.* in 2019 stated that there are limited data to assess the efficacy or safety profile of prophylactic mesh in the emergency setting^7,48^. Finally, even in the most recent papers by Jairam *et al.* (2020), Tansawet *et al*. (2020), and Depuydt *et al.* (2021), results were expressed as a mixture of elective and emergency surgery^12,49,50^. Diener *et al.* stated in 2010 how additional data and evidence were necessary to define the optimal closure method in the emergency setting, considering promising suture materials and strategies—such as prophylactic mesh augmentation. The current systematic review and meta-analysis focused on emergency laparotomy subgroup analysis and, to the best of our knowledge, is the first piece of evidence about this topic.

Currently, the updated guidelines for the closure of the abdominal wall incisions from the European and American Hernia Societies also consider the role of the mesh augmentation technique^1,2^. Statements by Deerenberg *et al.* confirm how prophylactic mesh augmentation after midline laparotomy can be taken into consideration to reduce the risk of IH. The quality of evidence of these statements is low and the strength of recommendation is weak. In addition, even these guidelines consider only elective midline laparotomy, because data on mesh augmentation in emergency settings are considered heterogeneous and limited without the possibility of a recommendation.

Further research and evidence are needed to identify definitive conclusions about the role of mesh augmentation in patients undergoing emergency midline laparotomy and to define subgroups of patients who might benefit the most from this technique: ongoing trials and this analysis follow this direction with a focus on the emergency population.

The PROPHYBIOM study (NCT 04681326) aims to evaluate the impact of a swine dermal collagen prosthesis implanted preperitoneally as a prophylactic procedure to prevent abdominal wall dehiscence in settings of urgency/emergency with contaminated/infected field^51^.

The ‘Preemer trial’ (NCT 04311788) is a multicentre, double-blind, RCT comparing a mesh group (retro rectus prophylactic self-gripping mesh) and a control group (4:1 small stitch closure by continuous monofilament suture) in case of emergency midline laparotomy for any gastrointestinal reason.

Finally, another ongoing study promoted by the Hospital del Mar in Barcelona (NCT 04808063) has the target to identify an algorithm to select patients who will benefit from prophylactic mesh application after midline laparotomy in emergency surgery.

The present study relies on a rigorous methodology and robust statistics. All studies about prophylactic mesh augmentation techniques in the closure of abdominal midline incisions, both in elective and emergency settings, were selected. Compared with similar previous systematic reviews and meta-analyses, the present research is based on a selection of studies ruling out overlapping series and considering only RCTs with the highest number of patients in the literature. Besides, to the best of our knowledge, this is the first meta-analysis about this topic that reports specific and significant results regarding prophylactic mesh positioning outcomes in the setting of urgent and emergency surgery.

However, several limitations need to be highlighted. First, the small number of studies included, especially in the setting of emergency surgery—this also limited the number of patients evaluated in the emergency surgery subgroup analysis. These results therefore need to be interpreted with caution. Furthermore, another question that remains unanswered is which type of mesh and surgical technique should be used in prophylactic mesh augmentation. In this regard, no subgroup analysis in the current study could be performed due to a critical heterogeneity of data, and the type of mesh and surgical technique could not be considered as relevant variables for the outcomes. Future perspectives should consider all these conditions as a target to identify the optimal technique and mesh choice to decrease IH after midline laparotomy, especially in emergency settings.

The recommended closure technique of abdominal midline incisions still has some unclear aspects despite evidence and guidelines reporting recent improvements: the high rate of IH and wound dehiscence, particularly in the setting of emergency surgery, is a challenging surgical problem resulting in significant morbidity. Prophylactic mesh placement is a technique of great interest with promising results in many RCTs; on the other hand, some surgeons are still concerned about the risk of infection, complications, and increased surgical times when a mesh is placed to reinforce abdominal wall closure.

According to data from this meta-analysis, prophylactic mesh placement could be considered a promising surgical technique to reduce IH rate in both elective and emergency surgery settings. The current results underline a significant decrease in the risk of IH at 1-, 2-, 3-, and 4-year follow-up, including emergency surgery patients. There is a trend towards a higher rate of wound dehiscence, wound complications, and seroma following mesh placement but this was not significant.

Further trials are needed to identify the ideal type of mesh and technique for mesh positioning, but surgeons should consider prophylactic mesh augmentation to decrease the IH rate, especially in patients at high risk of fascial dehiscence and even in emergency settings.

## Supplementary Material

zrad060_Supplementary_Data

## Data Availability

The authors declare that the data supporting the findings of this study are available within the article and its supplementary files.
